# Apixaban anti-Xa level monitoring in treatment of acute upper extremity deep vein thrombosis for patient on chronic hemodialysis: a case report

**DOI:** 10.1186/s12959-021-00277-8

**Published:** 2021-04-01

**Authors:** Guillaume Roberge, Philip Stephen Wells

**Affiliations:** 1 Department of General Internal Medicine, Centre Hospitalier Universitaire de Québec, Université Laval, Hôpital Saint-François d’Assise, 10 rue de l’Espinay, Québec, G1L 3L5 Canada; 2 Department of Medicine, University of Ottawa, the Ottawa Hospital Research Institute, Ottawa, Ontario Canada

**Keywords:** Venous thromboembolism, Apixaban, Anti-Xa, Dialysis, end-stage renal disease

## Abstract

**Background:**

Patients on dialysis are at higher risk of major bleeding and recurrent thrombosis creating acute venous thromboembolism (VTE) treatment challenges. DOACs represent an interesting option but there are concerns of bioaccumulation and increased bleeding risk. Anti-Xa trough levels may be used to monitor for bioaccumulation but there is little data.

**Case presentation:**

We describe a case, a 51 yo female, 36 kg on hemodialysis with a provoked acute upper extremity deep vein thrombosis in whom body habitus and calciphylaxis contraindicated the use of standard therapy. She received apixaban 2.5 mg twice daily for 6 weeks. The apixaban anti-Xa trough levels were measured weekly 12 h after the morning dose and ranged from 58 to 84 ng/mL, similar to expected levels with normal renal function. There were no adverse events in the 3 months follow-up.

**Conclusions:**

We saw no evidence of bioaccumulation indicating a potential role for low dose apixaban for acute VTE in dialysis patients.

## Background

Initiation of anticoagulation in patients with end stage renal disease (ESRD) and on chronic hemodialysis, who develop a venous thromboembolic event (VTE), is challenging since the rate of recurrent thrombosis and major bleeding are increased [1]. Ideally, when judged safe, treatment in such patients would avoid inpatient admission as for most others with acute VTE [2, 3]. Warfarin use in patients under chronic hemodialysis is associated with calciphylaxis and a high bleeding rate, while INR monitoring shows unsatisfactory time in therapeutic range [4–6]. Adjusted doses of low molecular weight heparin (LMWH) with anti-Xa monitoring is less than ideal due to parenteral administration and the risk of sub optimal management considering there is no standard LMWH protocol in patients with ESRD [7]. Direct oral anticoagulants (DOACs) represent an interesting option but there is concern over bioaccumulation which could increase bleeding risk [8–14]. Despite the absence of a standardized therapeutic range, anti-Xa trough levels are often measured to monitor potential bioaccumulation and thus, used for safety surveillance [15]. Apixaban has a potential favorable profile in ESRD and is allowed in this population for stroke prevention in atrial fibrillation [16]. However, for VTE treatment, apixaban in ESRD or dialysis is not yet recommended [2, 17]. Nevertheless, in specific circumstances, apixaban might be the best option for VTE management in ESRD and dialysis. We describe a case of a 51 yo female, 36 kg, on chronic hemodialysis with a provoked acute upper extremity deep vein thrombosis treated with apixaban.

## Case presentation

**Fig. 1:**
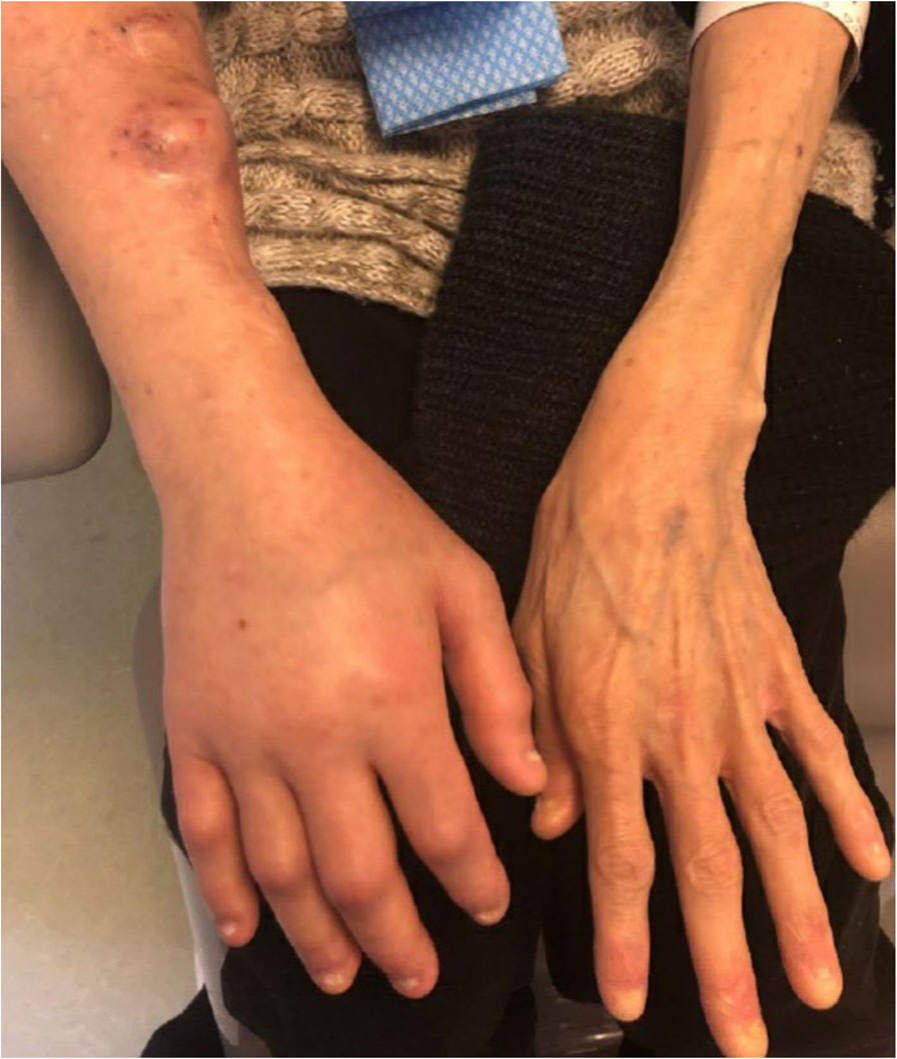
Right arm edema

A 51-year-old woman was seen in consultation for right arm discomfort and edema (Fig. 1). She had a past medical history of multiple bronchiectasis infections in context of congenital ectodermal dysplasia. Recurrent sepsis led to acute kidney injuries and eventually to anuric ESRD. She required hemodialysis 3 times a week, currently through using a right radiocephalic arteriovenous (AV) fistula, for almost 25 years. A renal transplant had failed immediately, 15 years prior. She had a right brachiocephalic vein stenosis, previously stented, and a suspicion of breast calciphylaxis diagnosed 3 years earlier. Her weight was 36 kg (BMI of 12) and she had negligible subcutaneous fat.

A right upper extremity ultrasound revealed a segmental nonocclusive thrombus in the lower part of the right internal jugular vein (IJV). She then had an angiogram and was diagnosed with a severe brachiocephalic vein in-stent stenosis and a moderate stenosis along the juxta-anastomotic cephalic vein fistula. Balloon dilatations of both stenosis were done but the intervention on the stent was complicated by a small focal contained rupture, which was again balloon dilated to ensure hemostasis. Soon after the intervention, the patient was seen at the thrombosis clinic. She reported improvement of her right arm symptoms and the initially described right nonocclusive IJV thrombosis was thought to be chronic. Surveillance by repeat ultrasound 1 week later without anticoagulation was chosen as initial management.

The next ultrasound reported interval progression of the known thrombus in the IJV (now occlusive) and nonocclusive extension into the subclavian vein. The stent was still patent. The patient was reporting worsening pain and swelling over the right side of her neck and her right arm for the last week. As such we diagnosed an acute right upper extremity deep vein thrombosis, most likely provoked by the initial flow disturbance caused by the in-stent stenosis and thereafter by the angiographic intervention.

She was treated as an outpatient with apixaban 2.5 mg twice daily for 6 weeks. Apixaban was taken at 10 am and 10 pm, while dialysis treatments were scheduled at 6 pm. At her 1 week follow up visit, the pain and swelling were improved. Over 4 weeks, apixaban anti-Xa levels were measured on dialysis days 8 h (i.e. 6 pm, before dialysis) and 12 h after the morning dose (i.e. trough levels, obtained at 10 pm, after dialysis and before the next dose). Anti-Xa were obtained using a CS-2000i (Siemens) analyzer with Innovance heparin reagent (Siemens) and calibrated by a STA®-Apixaban Calibrator kit (Diagnostica Stago, working range: 20–500 ng/mL). Surveillance ultrasound done 1 and 5 weeks after treatment initiation revealed stability of the upper extremity deep vein thrombosis and patency of the stent.

Over 4 weeks, the anti-Xa trough levels ranged from 58 to 84 ng/mL, while levels obtained before dialysis, i.e. 8 h after the morning dose, were 92–135 ng/mL. Monitoring was not continued up to the end of the treatment considering there was no trend of anti-Xa levels to increase overtime. There was no concomitant medication with significant apixaban interactions and liver function was normal. These range of anti-Xa levels are similar to what would be expected as a trough level in patients with preserved renal function. There were no adverse events in the 3 months after anticoagulation initiation.

## Discussion

This patient with an acute upper extremity deep vein thrombosis was considered with a high risk of bleeding because of her weight of 36 kg and her ESRD under chronic dialysis. VTE needed to be treated to prevent pulmonary embolism but also to preserve her right radiocephalic AV fistula function, as interrupted hemodialysis treatment is associated with increased hospitalizations and mortality [18]. Due to a lack of subcutaneous fat for safe injections and her history of calciphylaxis, we were reluctant to use low molecular weight heparin and warfarin [6, 19].

Apixaban was chosen mainly by extrapolation of observational data from atrial fibrillation literature [16, 20]. A network meta-analysis suggest apixaban might be associated with a lower major bleeding risk compared to warfarin and others DOACs in dialysis [21]. However, there is no randomized controlled trial yet validating such results [22]. Data about acute VTE treatment with apixaban in dialysis are more scarce and limited, but seems reassuring [23–27]. There are also some favorable small cases series in which dialysis patients with calciphylaxis were treated with apixaban for VTE treatment [28, 29]. None report anti-Xa monitoring in these scenarios and variable doses were used.

The optimal apixaban dose in hemodialysis is unknown, but we used the lowest dose to avoid potential bioaccumulation and bleeding in the context of ESRD and an extremely low body weight. A pharmacokinetic study looked at potential bioaccumulation of apixaban at steady state in 7 hemodialysis patients. Anti-Xa trough level after 2.5 mg twice daily dose measured on day 1 and 8 showed an increase of trough level from 45 to 132 ng/mL, but with same drug exposure (i.e. area under the concentration-time curve) as patients with preserved kidney function taking a 5 mg twice daily dose. Only 4% of the medication was said to be removed by dialysis. However, apixaban 5 mg given twice daily in hemodialysis led to significant bioaccumulation [8]. The relationship of anti-Xa levels and outcomes with DOACs are unknown since no outcome study has been based on anti-Xa levels. Only one case report mentions monitoring anti-Xa levels with apixaban in a dialysis patient. However, it was in context of atrial fibrillation and the anti-Xa levels were measured without apixaban calibration, meaning it was impossible to interpret [30].

We also aimed for a shorter 6-week treatment course considering uncertainty about risk-benefit balance in this patient. The optimal duration of treatment for a provoked VTE in ESRD and on dialysis patients is unknown. Management needs to be adapted in exceptional circumstances where balance between risk and benefit is precarious.

The apixaban anti-Xa trough levels obtained over 4 weeks using the 2.5 mg twice daily dose (58 to 84 ng/mL) are in line with the recognized on-treatment range in patients with preserved renal function [17]. Since apixaban is minimally impacted by dialysis and the anti-Xa levels obtained before it would still have been reassuring as potential trough, it is unlikely that elimination of apixaban by hemodialysis has resulted in false reassuring anti-Xa trough levels. The outcomes in the 3 months after anticoagulation initiation were favorable. However, caution is advised since safety and efficacy of DOACs for VTE treatment in such patients (i.e. in hemodialysis with low body weight, calciphylaxis and with the presence of AV fistula and a brachiocephalic vein stent) has not been established. A study addressing the question in this high risk and too often excluded population is greatly needed. As far as we know, this is the first publication of apixaban anti-Xa level monitoring in treatment of acute VTE for a patient on chronic hemodialysis.

## Conclusion

In conclusion, we saw no evidence of bioaccumulation. This indicates a potential role for low dose apixaban for acute venous thromboembolism in patients on dialysis.

## Data Availability

Data sharing is not applicable to this article as no datasets were generated or analysed during the current study.

## Abbreviations

ESRD: End stage renal disease
VTE: Venous thromboembolic event
LMWH: Low molecular weight heparin
DOAC: Direct oral anticoagulant
AV: Arteriovenous
IJV: Internal jugular vein
